# Supplementary material for: One-dimensional Magnus-type platinum double salts
Source: Nat Commun. 2016 Jun 20;7:11950. doi: 10.1038/ncomms11950 (PMC4915127; doi:10.1038/ncomms11950)
Supplement: Supplementary Information — Supplementary Figures 1-16, Supplementary Tables 1-5, Supplementary Notes 1-4 and Supplementary References [file ncomms11950-s1.pdf]

Supplementary Information

Supplementary Figures

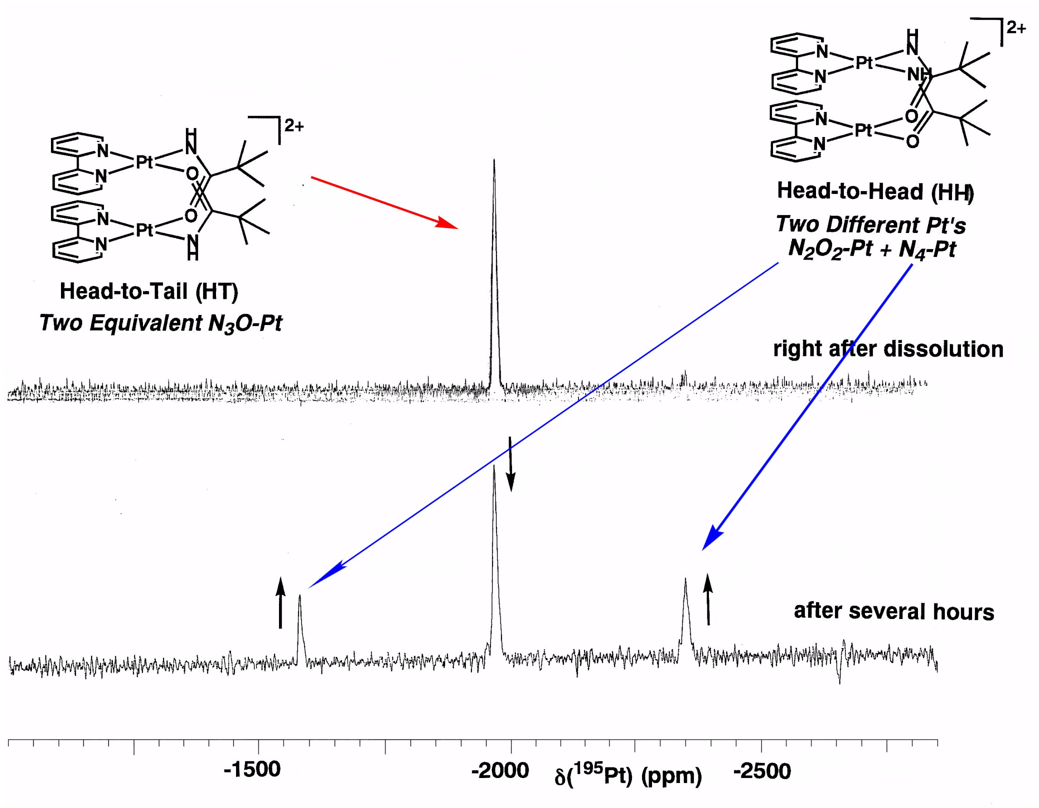

Supplementary Figure 1. ^{195}Pt NMR of the HT-to-HH isomerisation of **2**.

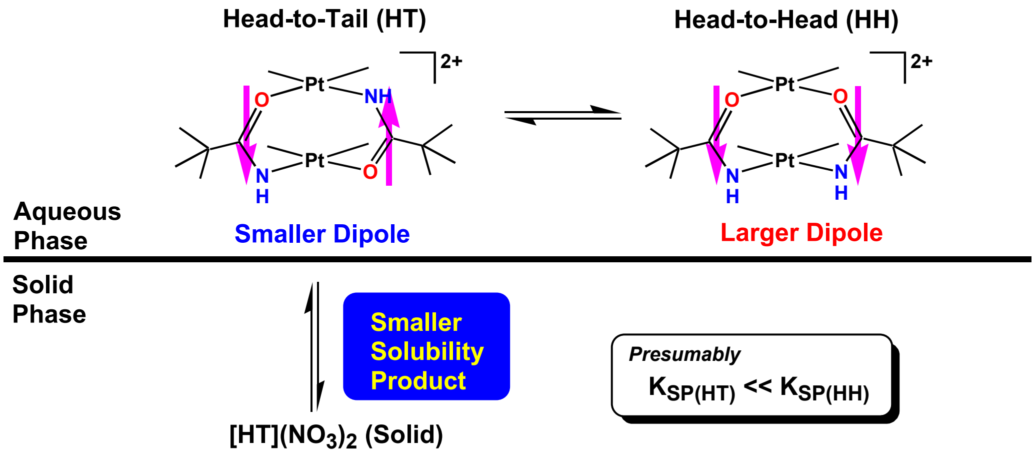

Supplementary Figure 2. The dipole moment in the solution phase motivates the HH isomerism.

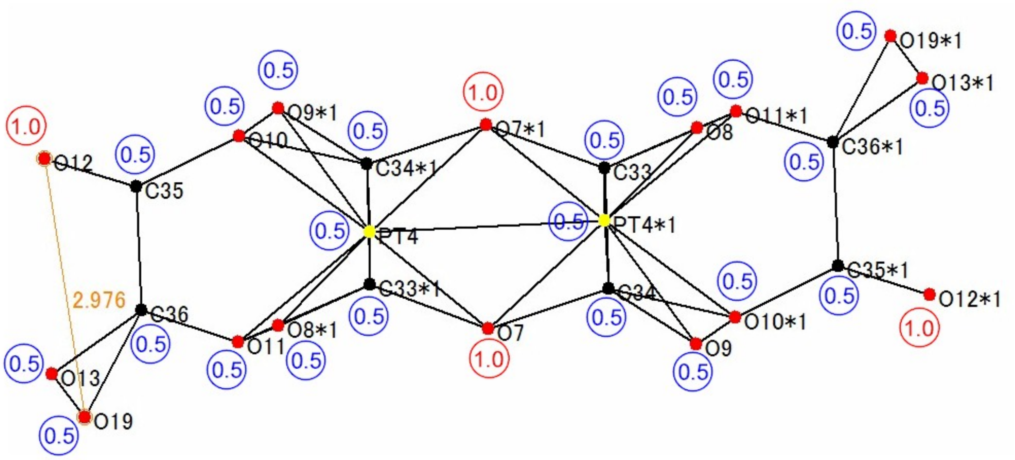

Supplementary Figure 3. Refinement of local Pt-geometry in **5**.

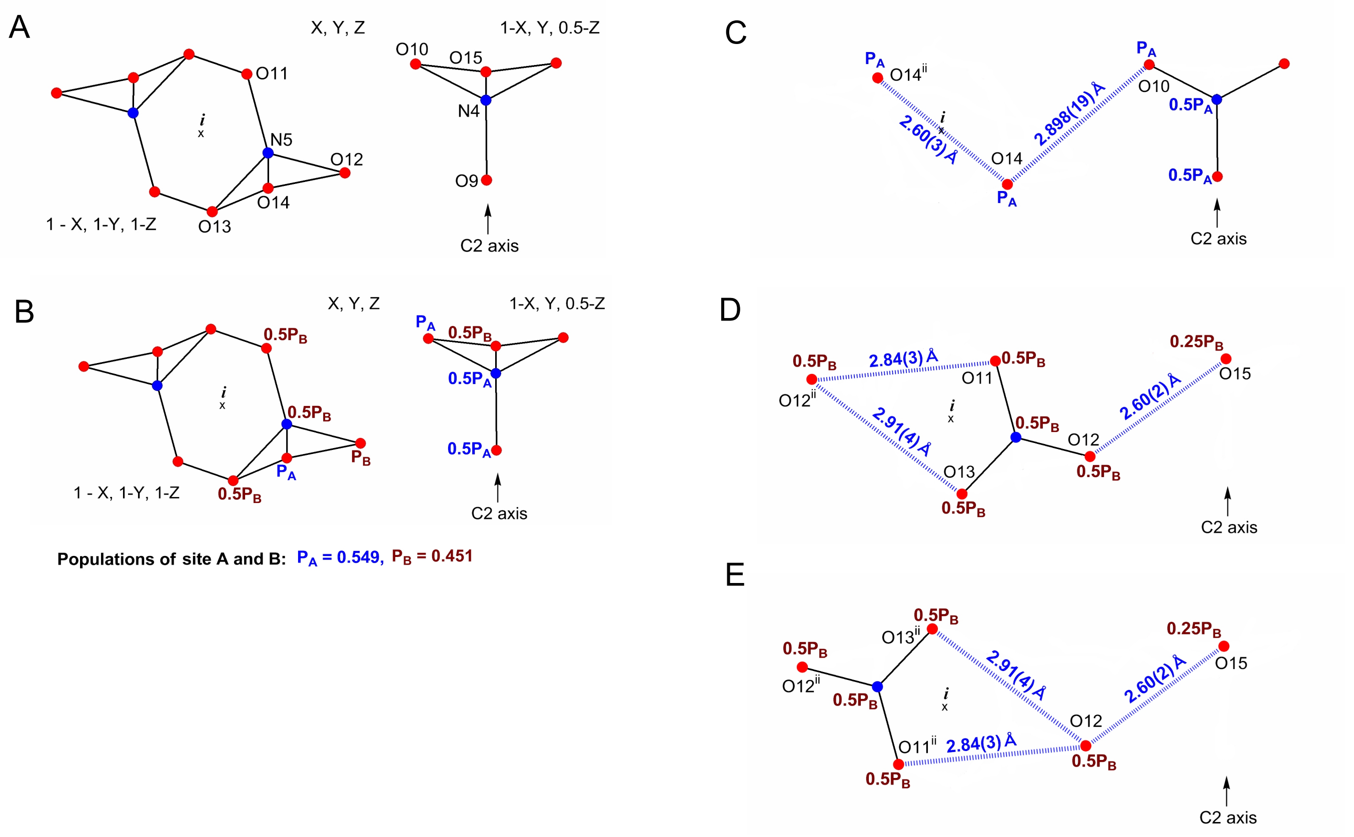

Supplementary Figure 4. Refinement of nitrate positions in **6**.

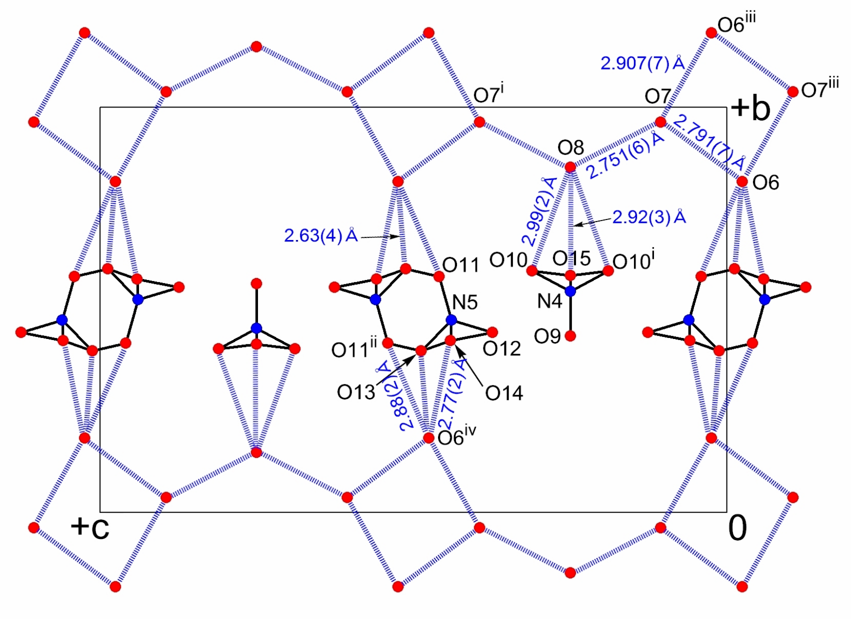

Supplementary Figure 5. Refinement of nitrate positions in **6** in the (200) plane.

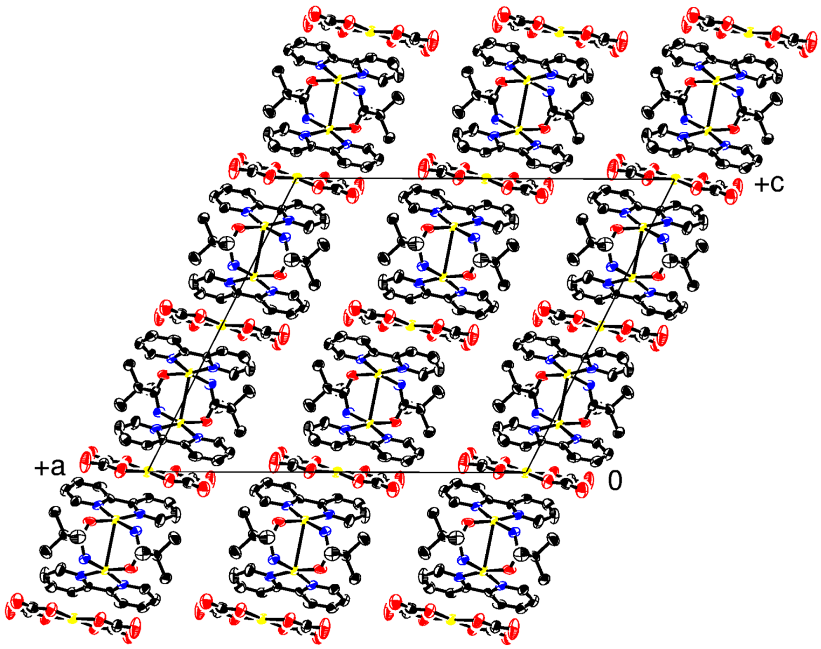

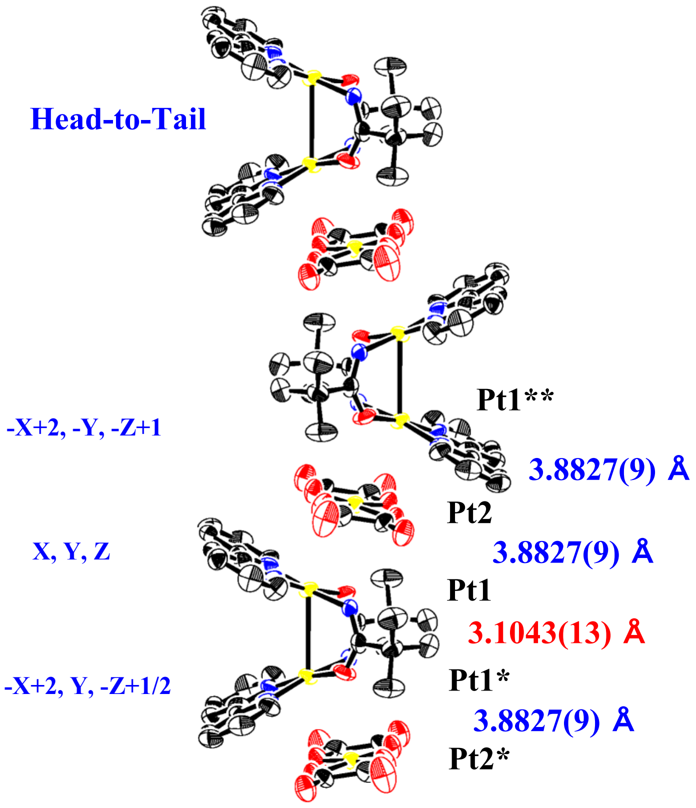

Supplementary Figure 6. ORTEP diagrams (50 % thermal ellipsoids) of [HT-$Pt_2(bpy)_2(\mu\text{-pivalamidato})_2$][$Pt(ox)_2$] (compound **3**), showing the packing diagram (top) and the alternating stack of complex cations and anions (bottom), where hydrogen atoms have omitted for clarity.

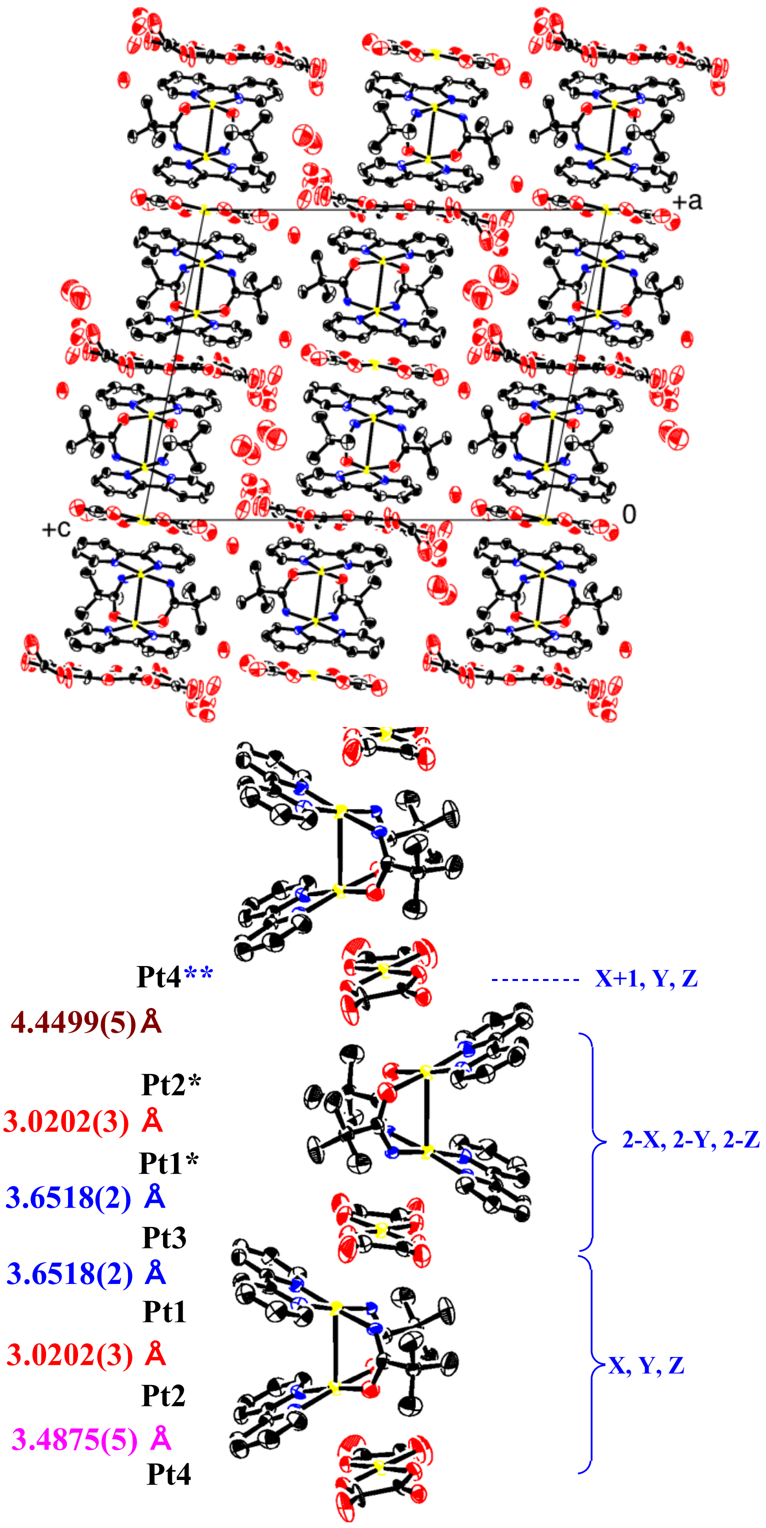

Supplementary Figure 7. ORTEP diagrams of [HH-Pt_2(bpy)$_2$(μ-pivalamidato)$_2$][Pt(ox)$_2$]·5.5H_2O (compound **4**), showing the packing diagram (top) and the alternating stack of complex cations and anions (bottom), where hydrogen atoms have omitted for clarity.

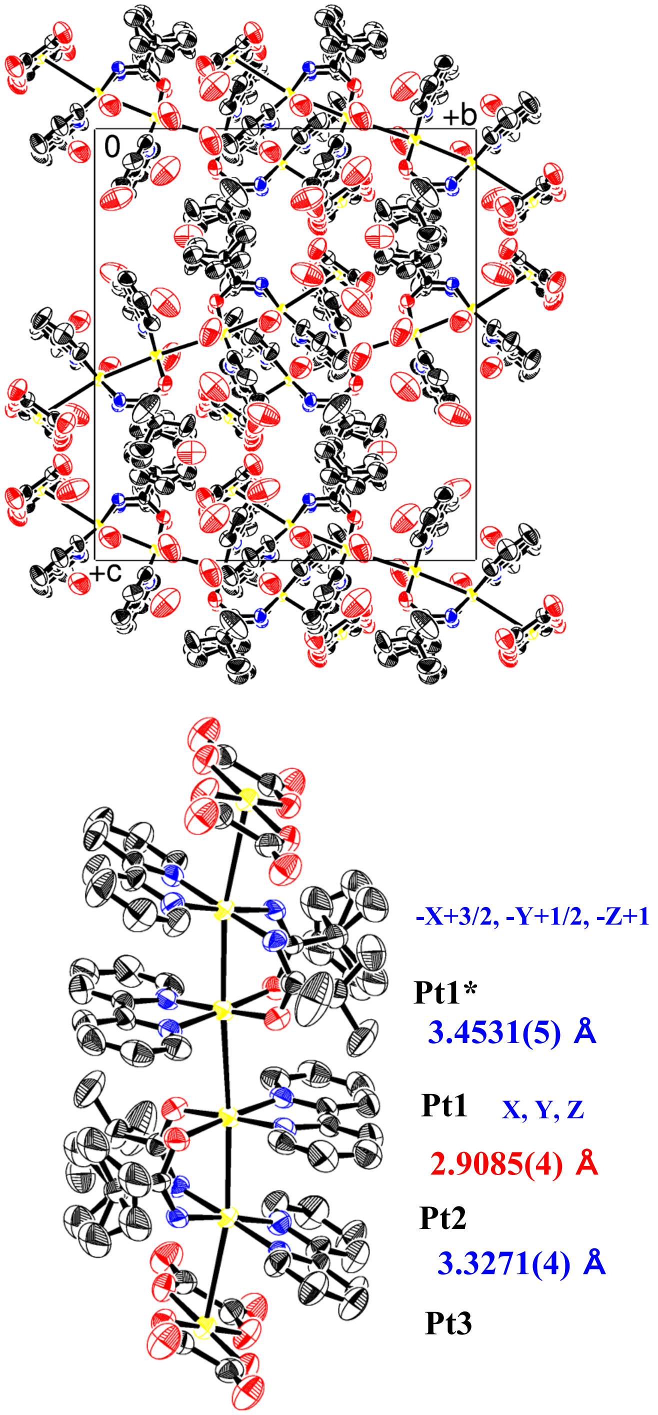

Supplementary Figure 8. ORTEP diagrams (50 % thermal ellipsoids) of $[HH\text{-}Pt_2(bpy)_2(\mu\text{-pivalamidato})_2]_2[Pt(ox)_2]_2{\cdot}8H_2O$ (compound **5**), showing the packing diagram (top) and the hexaplatinum chain structure of the complex (bottom), where hydrogen atoms have omitted for clarity.

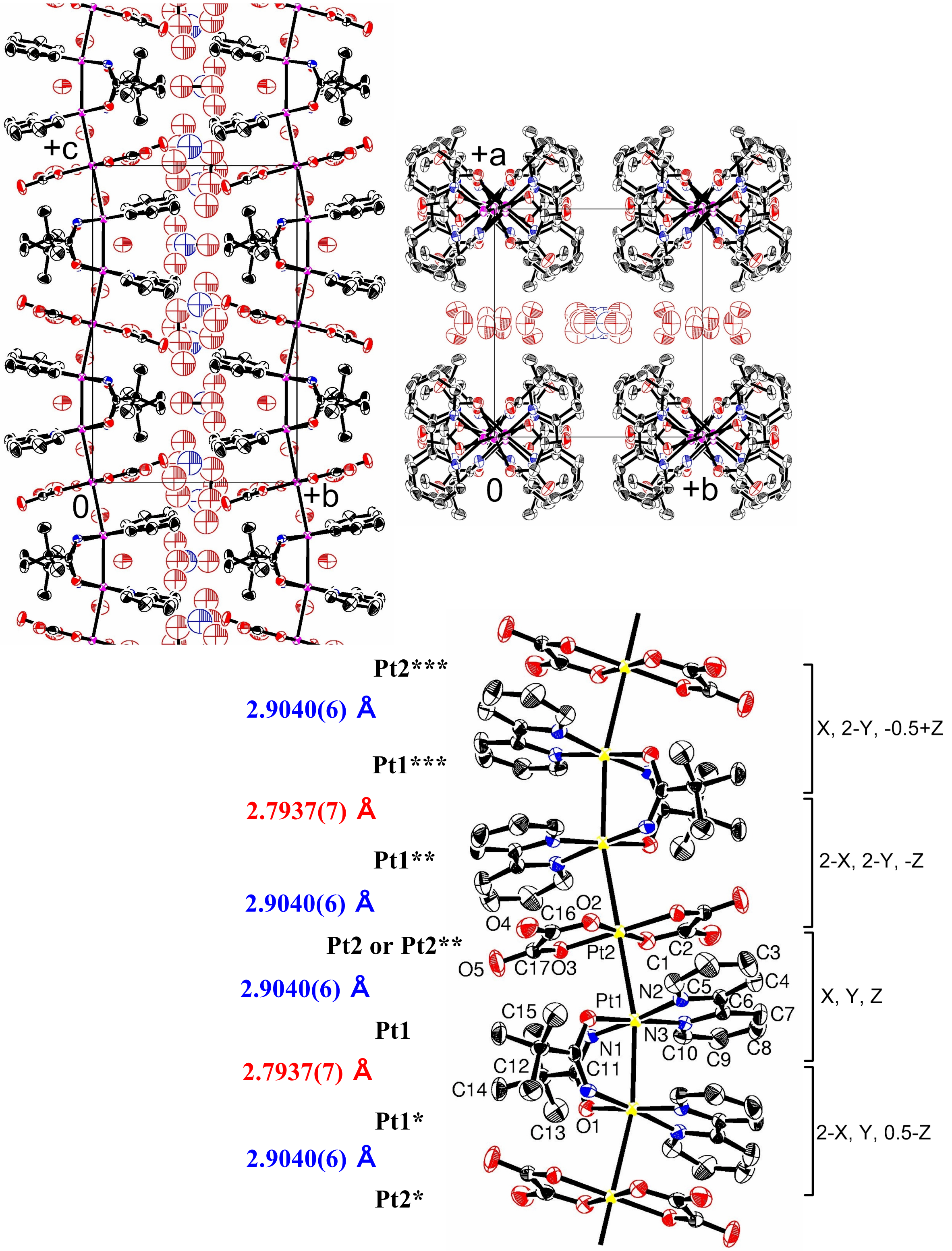

Supplementary Figure 9. ORTEP diagrams (50 % thermal ellipsoids) for the partially oxidized one-dimensional Pt(2.33+) chain structure of [HT-$Pt_2(bpy)_2$(μ-pivalamidato)$_2$][$Pt(ox)_2$] (compound **6**), showing the crystal packing view (top) and the cationic 1-D chain structure omitting the nitrate and solvent geometry (bottom), where hydrogen atoms have been omitted for clarity.

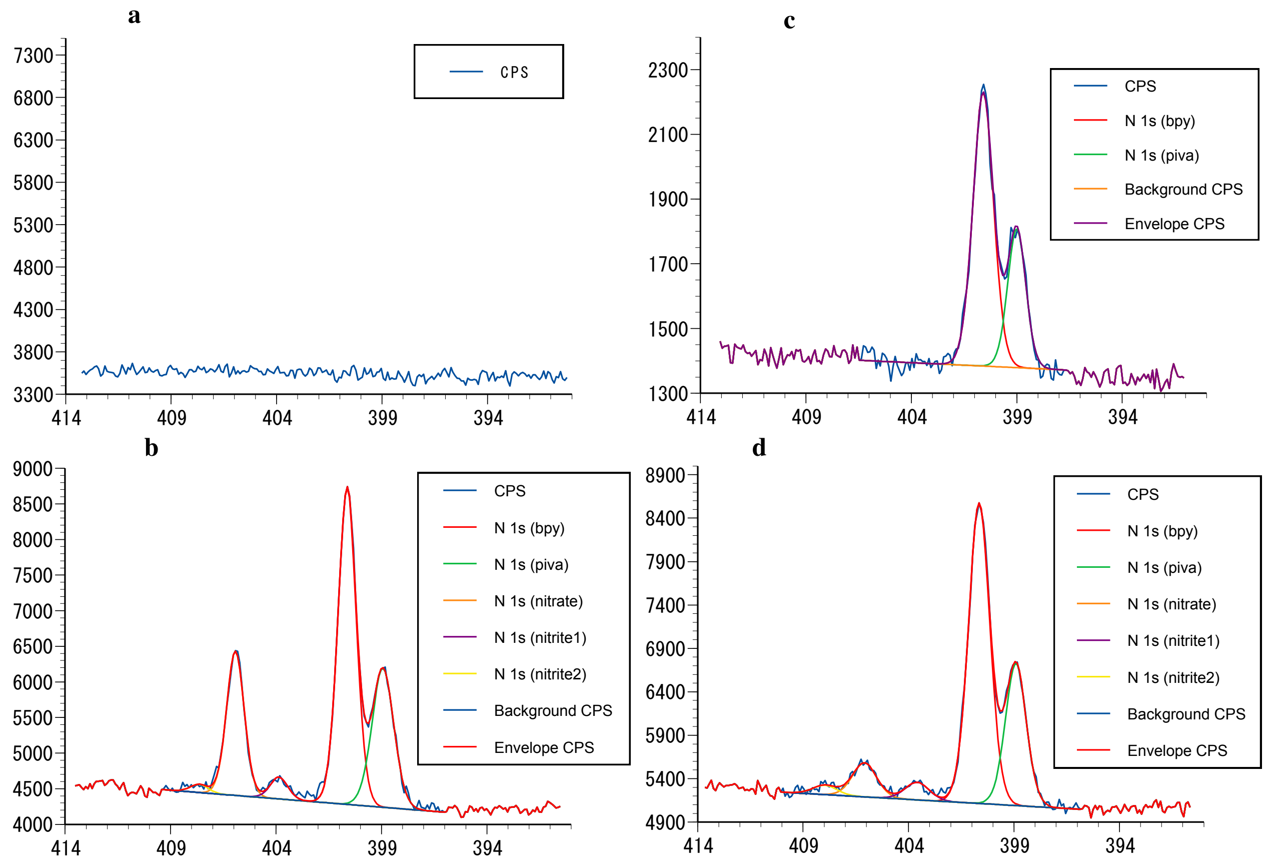

Supplementary Figure 10. X-ray photoelectron spectra at the N 1s region: (a) $K_2[Pt(ox)_2]$ (**1**); (b) [HT-Pt(II)$_2$(bpy)$_2$(μ-pivalamidato)$_2$]$(NO_3)_2 \cdot 5H_2O$ (**2**); (c) [HT-Pt(II)$_2$(bpy)$_2$(μ-pivalamidato)$_2$][Pt(II)(ox)$_2$] (**3**); (d) [HT-Pt$_2$(bpy)$_2$(μ-pivalamidato)$_2$][Pt(ox)$_2$]$(NO_3) \cdot 7H_2O$ (**6**) (Shimadzu AXIS ULTRA). Deconvolution was carried out using CasaXPS in which a linear type background was employed, the mixing ratio of Gaussian and Lorentzian functions was fixed at 70:30. The FWHM values for the apparently overlapped components at the nitrate/nitrite domain (402-409 eV) were constrained to be equal. The analytical data suggest that the number of nitrate per formula unit for **6** may roughly be considered as unity (see Tables S3, S4).

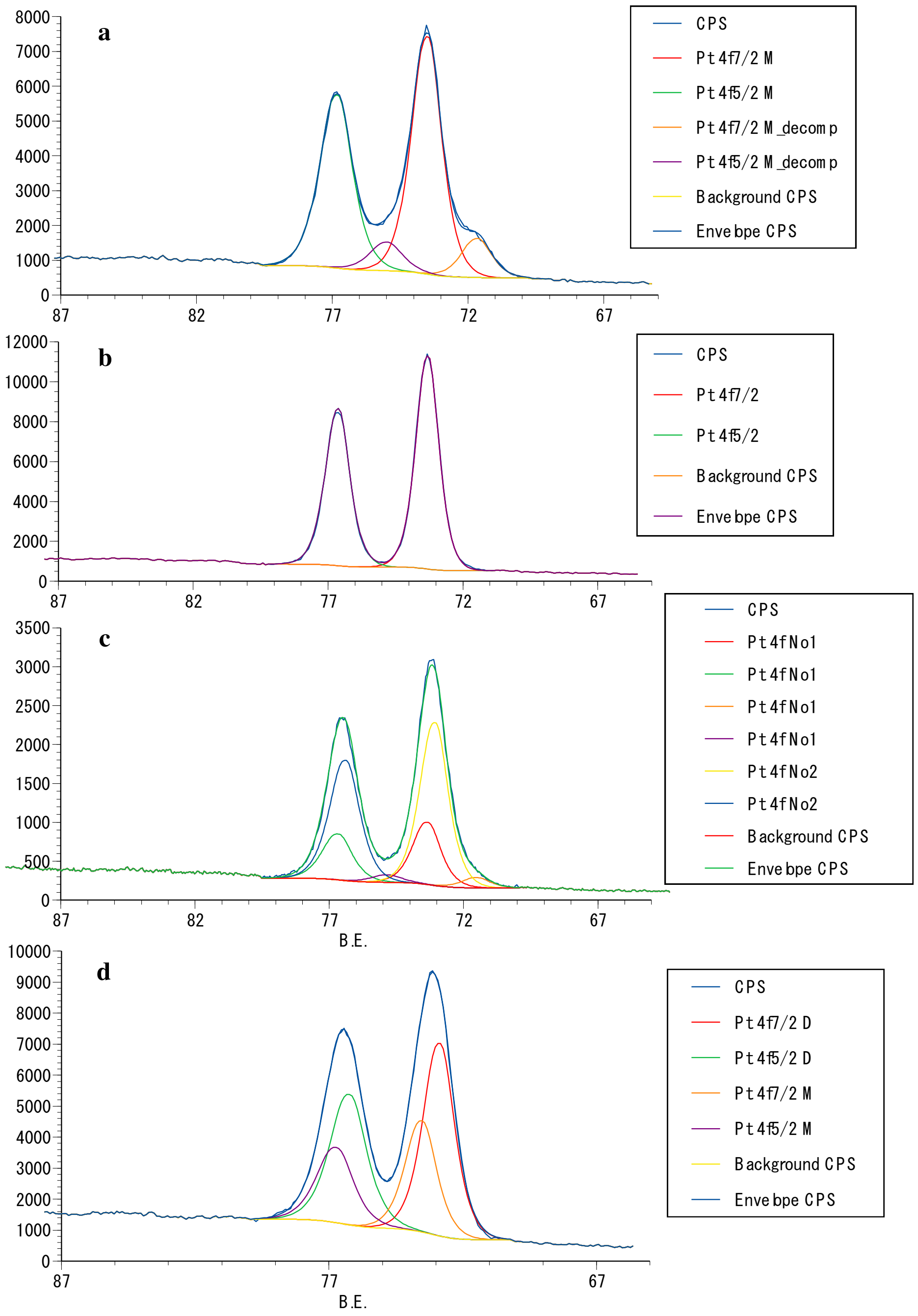

Supplementary Figure 11. X-ray photoelectron spectra at the Pt $4f_{7/2}$ and $4f_{5/2}$ region: (a) $K_2[Pt(ox)_2]$ (**1**); (b) [HT-Pt(II)$_2$(bpy)$_2$(μ-pivalamidato)$_2$]$(NO_3)_2 \cdot 5H_2O$ (**2**); (c) [HT-Pt(II)$_2$(bpy)$_2$(μ-pivalamidato)$_2$][Pt(II)(ox)$_2$] (**3**); (d) [HT-Pt$_2$(bpy)$_2$(μ-pivalamidato)$_2$][Pt(ox)$_2$]$(NO_3) \cdot 7H_2O$ (**6**) (Shimadzu AXIS ULTRA). Deconvolution was carried out using CasaXPS in which a Shirley type background was adopted, and the mixing ratio of Gaussian and Lorentzian functions was fixed at 70:30. In our deconvolution strategy, the overlapped $4f_{7/2}$ signals were constrained to possess a common FWHM parameter. The same constraint was applied to the $4f_{5/2}$ signals. Moreover, the area ratios of the $4f_{5/2}$ and $4f_{7/2}$ signals for each origin were constrained to have a theoretical value, 0.787. The results of deconvolution treatments are summarized in Supplementary Table 5.

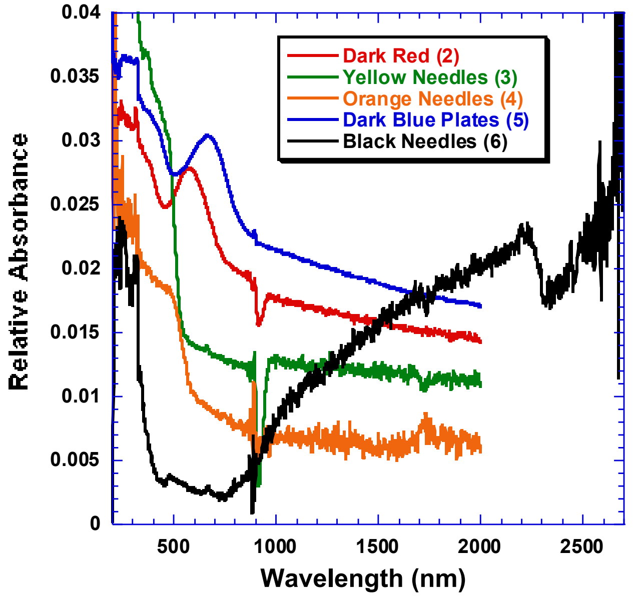

Supplementary Figure 12. Solid-state absorption spectra of compounds **2**-**6** observed by the Nujol mull method at room temperature. Each crystalline sample was finely ground in a liquid paraffin matrix and the resulting suspension was transferred into a quartz cell having an optical path length of 0.1 mm. The spectrum in the range 200-2700 nm was measured on a Shimadzu UV3150-K UV-Visible-NIR spectrophotometer. The observed broad spectral feature of **6** is similar to those observed for the partially oxidized one-dimensional complexes consisting of acetate- and propionate bridged dinuclear Pt 2.2+ units, $[Pt(2.2+)_2(NH_3)_4(\mu\text{-acetato})_2](NO_3)_{2.4}\cdot 2H_2O$ and $[Pt(2.2+)_2(NH_3)_4(\mu\text{ -propionato})_2](NO_3)_2(ClO_4)_{0.4}\cdot 2H_2O$, which were reported in Ref 32.

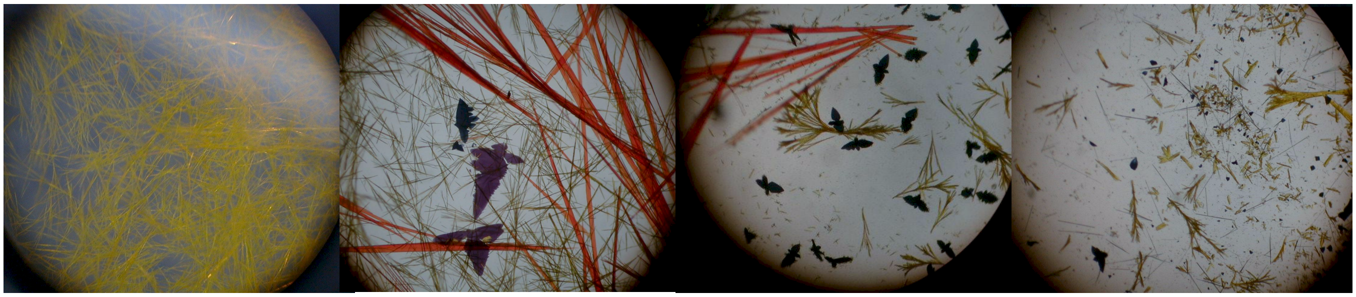

Supplementary Figure 13. Optical microscopy images of compounds **3**, **4**, **5** and **6.**

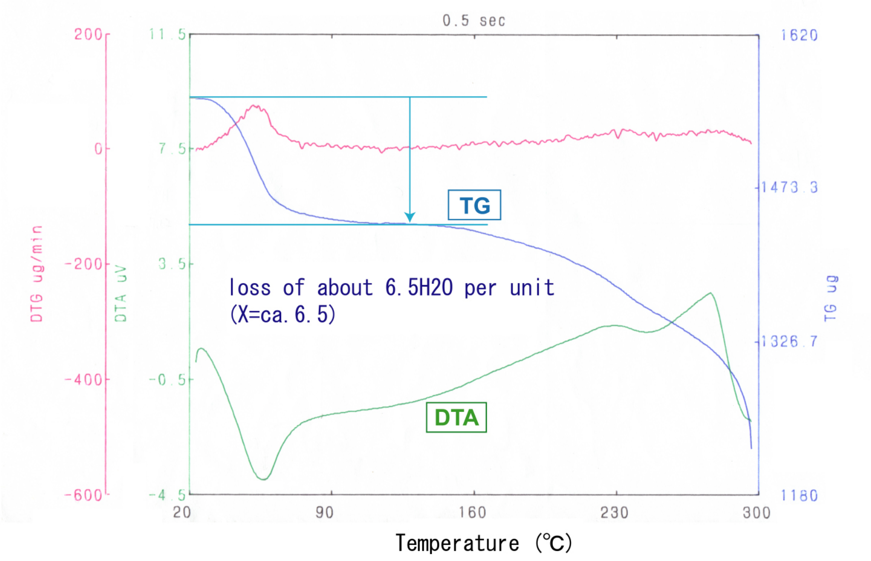

Supplementary Figure 14. Thermogravimetric analysis of compound **6**, confirming the presence of approximately 7 water molecules per unit cell.

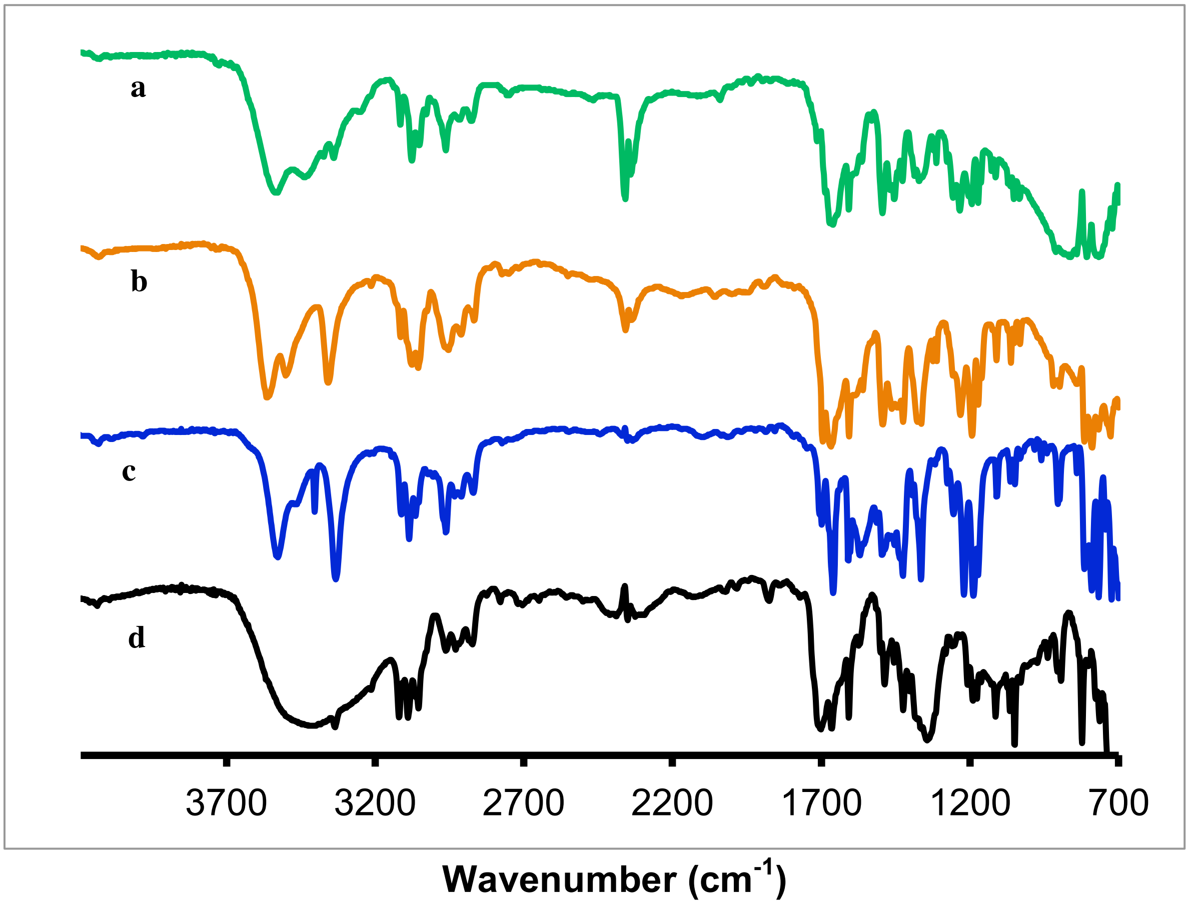

Supplementary Figure 15. Single-crystal IR spectra observed at room temperature; (a) [HT-Pt(II)$_2$(bpy)$_2$(μ-pivalamidato)$_2$][Pt(II)(ox)$_2$] (**3**); (b) [HH-Pt(II)$_2$(bpy)$_2$(μ-pivalamidato)$_2$][Pt(II)(ox)$_2$]·5.5H_2O (**4**); (c) [HH-Pt(II)$_2$(bpy)$_2$(μ-pivalamidato)$_2$]$_2$[Pt(II)(ox)$_2$]$_2$·8H_2O (**5**); (d) [HT-Pt$_2$(bpy)$_2$(μ-pivalamidato)$_2$][Pt(ox)$_2$](NO_3)·7H_2O (**6**), showing the appearance of a broad 1400-cm^{-1} peak derived from the nitrate anion (NO_3^-) involved in **6**.

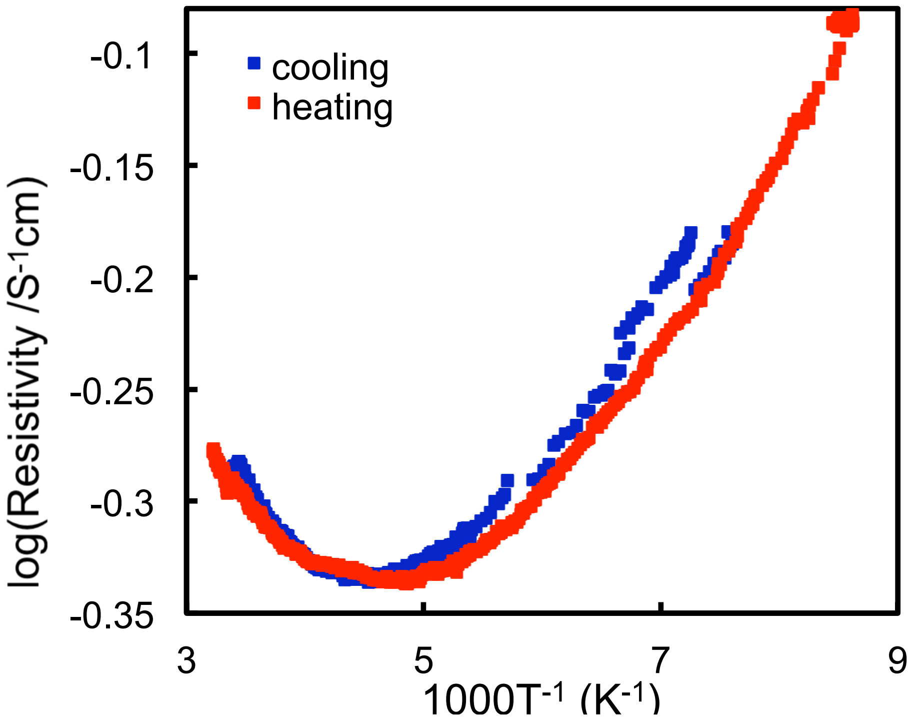

Supplementary Figure 16. Arrhenius plot of the log conductivity vs. inverse absolute temperature converted from the conductivity vs. temperature shown in Figure 4e.

Supplementary Tables

Supplementary Table 1. Results of CHN analysis for **3**-**6**.[a]

Compound	nuclearity	C (%) Calcd. (Found)	H (%) Calcd. (Found)	N (%) Calcd. (Found)
HT-$Pt_3O_{10}N_6C_{34}H_{36}$ (**3**)	pseudo-1-D	32.06 (32.02)	2.85 (2.65)	6.60 (6.22)
HH-$Pt_3O_{10}N_6C_{34}H_{36}\cdot 4.4H_2O$ (i.e., **4** – $1.1H_2O$)[b]	pseudo 1-D	30.18 (30.21)	3.34 (3.25)	6.21 (5.97)
[HH-$Pt_3O_{10}N_6C_{34}H_{36}]_2\cdot 4.6H_2O$ (i.e., **5** - $3.4H_2O$)[b]	hexanuclear	31.04 (31.34)	3.11 (2.83)	6.39 (6.03)
[HT-$Pt_3O_{10}N_6C_{34}H_{36}](NO_3)\cdot 7H_2O$ (**6**)	1-D	27.93 (28.12)	3.45 (3.22)	6.71 (6.46)

[a]$Pt_3O_{10}N_6C_{34}H_{36}$ = $[Pt_2(bpy)_2(\mu\text{-pivalamidato})_2][Pt(ox)_2]$.

[b]These samples readily lose crystal solvents (water molecules) in air.

Supplementary Table 2. Crystallographic Data for **3-6**.

	compound **3**	compound **4**	compound **5**	compound **6**
formula	$C_{34}H_{36}N_6O_{10}Pt_3$	$C_{34}H_{47}N_6O_{15.5}Pt_3$	$C_{68}H_{88}N_{12}O_{28}Pt_6$	$C_{34}H_{50}N_7O_{20}Pt_3$
fw	1273.96	1373.05	2692.04	1462.08
color, habit	yellow, needle	orange, plate	dark blue, plate	black, needle
crystal size, mm	0.45x0.038x0.01	0.2x0.1x0.03	0.02x0.02x0.005	0.643x0.069x0.039
crystal system	Monoclinic	Monoclinic	Monoclinic	Monoclinic
space group	C2/c	P 2_1/n	C 2/c	P2/c
a, Å	23.042(5)	18.7199(6)	22.9917(8)	12.158(2)
b, Å	9.2011(19)	9.6901(3)	17.8912(6)	10.943(2)
c, Å	20.049(4)	23.7454(8)	20.7881(7)	16.944(3)
a, deg	90.00	90.00	90.00	90.00
b, deg	116.718(4)	101.185(1)	102.477(1)	93.548(4)
g, deg	90.00	90.00	90.00	90.00
V, $Å^3$	3796.7(13)	4225.5(2)	8349.2(5)	2250.0(7)
Z	4	4	4	2
F(000)	2384	2604	5088	1394
d_{calc}, g/cm^3	2.229	2.158	2.142	2.158
μ(Mo Kα), mm^{-1}	11.089	9.981	10.098	9.387
trans. coeff.	0.224352 - 0.694171	0.40331 - 0.69484	0.424991 - 0.694844	0.2404 - 0.6922
abs. correction	multi-scan[a]	multi-scan[a]	multi-scan[a]	gaussian[b]
T, K	296(2)	200(2)	296(2)	296(2)
radiation, Å	0.71073	0.71073	0.71073	0.71073
θ range, deg	2.27 < θ < 27.75	2.22 < θ < 30.07	1.65 < θ < 30.05	1.68 < θ < 27.52
index ranges (h)	-25 < h < 29	-26 < h < 24	-32 < h < 24	-15 < h < 15
index ranges (k)	-11 < k < 11	-13 < k < 13	-25 < k < 22	-6 < k < 14
index ranges (l)	-26 < l < 23	-33 < l < 30	-28 < l < 29	-21 < l < 21
reflns measd	11724	29259	28567	14637
uniq reflns	4296	12295	12169	5126
R(int)	0.0903	0.0447	0.0725	0.0386
data/restraints/params	4296/0/244	12295/0/599	12169/0/525	5126/15/292
R1	0.0596	0.0331	0.0372	0.0249
wR2	0.1499[c]	0.0678[d]	0.0673[e]	0.0512[f]
GOF	0.905	0.911	0.815	0.873
max/mean shift/esd	0.001/0.000	0.003/0.000	0.002/0.000	0.001/0.000
max/min diff. peaks	3.056/-2.523	1.368/-0.854	1.136/-1.071	1.588/-0.725

[a] SADABS; Sheldrick, 1996 (ref. S2).
[b] XPREP in SAINT; Bruker, 2001 (ref. S3).
[c] Weighting Scheme: $1/w = [\sigma^2(F_o^2)+(0.0758P)^2+0.0000P]$ where $P=(F_o^2+2F_c^2)/3$.
[d] Weighting Scheme: $1/w = [\sigma^2(F_o^2)+(0.0235P)^2+0.0000P]$ where $P=(F_o^2+2F_c^2)/3$.
[e] Weighting Scheme: $1/w = [\sigma^2(F_o^2)+(0.0160P)^2+0.0000P]$ where $P=(F_o^2+2F_c^2)/3$.
[f] Weighting Scheme: $1/w = [\sigma^2(F_o^2)+(0.0162P)^2+0.0000P]$ where $P=(F_o^2+2F_c^2)/3$.

Supplementary Table 3. N 1s Binding Energies for Complex **1.**

	bpy	pivalamidate	nitrate		
	N 1s (bpy)	N 1s (piva)	N 1s (nitrate)	N 1s (nitrite1)	N 1s (nitrite2)
Binding Energy (eV)	400.63	398.93	405.93	403.86	407.64
FWHM (eV)	1.01	1.21	0.97	0.97	0.97
Area	4868	2557	2137	324	124
	4868	2557	2585		
molar ratio	4.00	2.00	2.00		

Supplementary Table 4A. N 1s Binding Energies for Complex **6** (run1).

	bpy	pivalamidate	nitrate		
	N 1s (bpy)	N 1s (piva)	N 1s (nitrate)	N 1s (nitrite1)	N 1s (nitrite2)
Binding Energy (eV)	400.75	399.00	406.26	403.77	408.27
FWHM (eV)	1.41	1.39	1.69	1.69	1.69
Area	4997	2291	695	326	186
	4997	2291	1206		
molar ratio	4.00	1.75	0.91		
molar ratio	4.58	2.00	1.04		
molar ratio	6.00		0.95		

Supplementary Table 4B. N 1s Binding Energies for Complex **6** (run2).

	bpy	pivalamidate	nitrate		
	N 1s (bpy)	N 1s (piva)	N 1s (nitrate)	N 1s (nitrite1)	N 1s (nitrite2)
Binding Energy (eV)	400.65	398.91	406.09	403.59	407.97
FWHM (eV)	1.18	1.16	1.34	1.34	1.34
Area	4424	2059	570	294	158
	4424	2059	1022		
molar ratio	4.00	1.77	0.87		
molar ratio	4.51	2.00	0.98		
molar ratio	6.00		0.91		

Supplementary Table 5. Pt 4f Binding Energies for **1**, **2**, **3** and **6**.

Compound	Binding Energies [FWHM] (eV)					
	Dimer Component		Monomer Component		Monomer Decomp.[a]	
	Pt $4f_{5/2}$	Pt $4f_{7/2}$	Pt $4f_{5/2}$	Pt $4f_{7/2}$	Pt $4f_{5/2}$	Pt $4f_{7/2}$
$K_2[Pt(II)(ox)_2]\cdot 2H_2O$ (**2**)			76.7989 [1.31]	73.4713 [1.21]	74.9625 [1.31]	71.6349 [1.21]
$[Pt(II)_2(bpy)_2(piva)_2]$-$(NO_3)_2\cdot 5H_2O$ (**1**)	76.6037 [0.99]	73.2761 [0.92]				
$[Pt(II)_2(bpy)_2(piva)_2]$-$[Pt(II)(ox)_2]$ (**3**)	76.378 [1.22]	73.0504 [1.15]	76.6543 [1.22]	73.3267 [1.15]	74.8179 [1.22]	71.4903 [1.15]
$[Pt(2.33+)_2(bpy)_2(piva)_2]$-$[Pt(2.33+)(ox)_2](NO_3)\cdot 7H_2O$ (**6**)[b]	76.2073 [1.49]	72.8405 [1.28]	76.6993 [1.49]	73.5094 [1.28]		

[a]Compounds **1** and **3** are insulators which is the major cause of their fast decomposition during the XPS measurements. In other words, compounds **2** and **6** are both electrical conductors preventing these materials to be charged during the measurements, which is considered as the major reason for the lack of peaks attributable to decomposed species.

[b]FWHM values estimated for compound **6** are still comparably narrow when compared to those observed for other homovalence copmouonds **1**-**3**. This is indicative of rapidly delocalized nature of the Pt(III) states along with the one-dimensional Pt chains, supporting the formal expression of Pt(2.33+) in the reported formula of **6**.

Supplementary Notes

Supplementary Note 1: Elemental Analysis

As summarized in Supplementary Table 1, only compounds **3** and **6** are stable upon exposure to air, while **4** and **5** lose part of water solvate while subjected for their elemental analysis. So the numbers of water solvate for **4** ($5.5H_2O$) and **5** ($8H_2O$) have been determined based on the results of their X-ray structure refinements.

Supplementary Note 2: Conductivity Measurements

The single-crystal electrical conductivity of **6**, parallel to the chain axis ($\sigma_{//}$), and its temperature dependence were measured based on the method of Montgomery (Montgomery, H. C., *J. Appl. Phys.* **1971**, *42*, 2971; Zeller, H. R., Beck, A. *J. Phys. Chem. Solids*, **1974**, *35*, 77.). Repeated experiments showed that the room-temperature conductivity ($\sigma_{//}$) varies in the range of 1-10 S/cm, where the electrical anisotropy was estimated as $\sigma_{//} / \sigma_{\perp} = 10^3 - 10^4$. A reversible metal-semiconductor transition was observed at around 210 K (Figure 4), in which the activation energy can be roughly estimated from the slope of the Arrhenius plot of the log conductivity vs. inverse absolute temperature (Supplementary Figure 16).

Supplementary Note 3: Structure of [HT-Pt(II)$_2$(bpy)$_2$(μ-pivalamidato)$_2$](NO$_3$)$_2$·5H$_2$O (2)

The major product in the synthesis of **2** has been confirmed to be the HT isomer in relatively high purity. This has been confirmed by ^{195}Pt NMR, Supplementary Figure 1, and powder X-ray diffractometry. The former study showed that the HT isomer undergoes a slow isomerization into the HH isomer over a few days in water at ambient temperature. As reported by O'Halloran and Lippard,[S1] the HT dimer is observed as a singlet in ^{195}Pt NMR, since it consists of two magnetically equivalent Pt atoms which are coordinated by three nitrogen atoms and an oxygen atom (two N_3O-coordinated Pt atoms exit). On the other hand, the HH dimer shows a doublet, since there are a N_4-coordinated and a N_2O_2-coordinated Pt atoms. The HT isomerism of **2** in the crystal is evident due to the crystallographic two-fold axis passing through the center of the diplatinum entity, which will be separately reported elsewhere combined with its unique solid-state physical properties.

Furthermore, about ten crystals of **2** picked up in a random manner were subjected onto the single-crystal X-ray diffractometry to confirm that there were only a single type of crystal obtained as a result of recrystallization of **2** from hot water. The selective deposition of **2** is attributable to the lower molecular dipole originated at the HT isomer in comparison with that of the HH isomer (the dipole moments originated along the two amidate N-O directions within a dimer unit are correlated with one another).

Supplementary Note 4: X-ray Crystallography Measurements and Software

Since the crystals of **3** and **6** are stable in air, they were mounted on glass fibers. As the crystals of **4** and **5** lose water solvate upon prolonged exposure to air, each crystal was sealed in a capillary glass tube with the mother liquor to avoid the loss of water solvate during the measurement. Measurements and structure analysis were carried out several times on several different crystals to ensure consistency. Diffraction data were collected on a Bruker Smart APEX CCD-detector X-ray diffractometer with graphite-monochromated Mo Kα radiation (λ = 0.71073 Å; sealed tube). The data collection was carried out at 296 K for **3**, **5**, and **6** and at 200 K for **4**. The data were corrected for Lorentz and polarization effects, and for absorption using the SADABS[S2] program for **3-5** and the analytical method implemented in the SAINT[S3] program for **6**. Structures were solved by the SHELXS-97[S4] program and were refined on F^2 by full-matrix least-squares using the SHELXL-97[S5] program. The KenX[S6] program was used to visualize molecules and to locate new atoms during the refinement procedures. PLATON[S7] program was also used to examine both the observed and difference Fourier maps, especially around the geometries where atoms are ambiguous due to the disorder phenomena. The teXsan[S8] program was used to generate ORTEP[S9] diagrams depicted in Figures S3-S6. All the reported distances and angles, including the results of mean-plane calculations, were calculated in SHELXL-97.[S5] Crystallographic data for **3-6** are summarized in Supplementary Table 2.

*Structure Refinement Details of [HT-Pt$_2$(bpy)$_2$(μ-pivalamidato)$_2$][Pt(ox)$_2$] (**3**)*

The asymmetric unit involves a half of the formula unit, where a two-fold axis passes through the midpoint of the HT dimer unit and an inversion center is located at the center of the $[Pt(ox)_2]^{2-}$ anion. The binding direction of O and NH of pivalamidate was unambiguously determined by comparing the results of least-squares calculations performed for two possible arrangements of the ligand. As described well for some relevant amidate-bridged platinum dimers,[S10-S14] a wrong combination generally gives an asymmetric feature with regard to the equivalent displacement parameters for the O and N atoms of bridging amidates, while an appropriate combination often gives a moderate balance in these parameters. The reported combination has been judged to be valid: U_{eq}(N1) = 0.040(3) and U_{eq}(O1) = 0.055(3), while the reverse combination clearly gives an asymmetric feature on these values: U_{eq}(N1 refined as O) = 0.061(3) and U_{eq}(O1 refined as N) = 0.0352(12). Moreover, the latter combination leads to clear increase in the final reliability factors ($R1[F^2 > 2\sigma(F^2)] = 0.0600$ and $wR2(F^2) = 0.1504$). As usual the case for platinum complexes, refinement of hydrogen atoms does not reach acceptable convergence and therefore all hydrogen atoms on the cationic moiety were refined using the standard riding model scheme implemented in SHELXL-97. Thus, all H atoms are located at their idealized positions (C-H(methyl) = 0.96 Å, C-H(bpy) = 0.93 Å, N-H(pivalamidate) = 0.86 Å), and included in the refinement in a riding model approximation, with U_{iso}(methyl H) = 1.5U_{eq}(bonded C), U_{iso}(aromatic H) = 1.2U_{eq}(bonded C), and U_{iso}(H of amidate N-H) =

1.2U_{eq}(bonded N). In the final difference Fourier synthesis, the highest peak (3.06 e/Å^3) was located at 0.98 Å of Pt1, while the deepest hole (-2.52 e/Å^3) was located at 0.92 Å from Pt1.

*Structure Refinement Details of [HH-$Pt_2(bpy)_2(\mu$-pivalamidato$)_2$][$Pt(ox)_2$]·5.5H_2O (**4**)*

The asymmetric unit consists of one formula unit. The atoms comprising the dimeric unit are all located in general positions with no disorder problem. In the asymmetric unit, two independent $[Pt(ox)_2]^{2-}$ anions are found with each having a half occupation factor, where one possesses an inversion center while the other is disordered around an inversion center. The Pt ion (Pt3) is located at an inversion center in the former, while the other (involving Pt4) is disordered around an inversion center in a rather complicated fashion as depicted in Supplementary Figure 7, where values in blue and red circles denote the site occupation factors of individual atoms.

O12 and O19 are supposed to be present in geometry where the $[Pt(ox)_2]^{2-}$ anion including atoms Pt4, C35, and C36 are not present. A slightly puzzling situation is the site occupation factor of O12 since the atom is supposed to behave as a water oxygen atom and an oxalate oxygen atom in an equal probability. For the water solvate geometries, O18A and O18B are supposed to be disordered over two sites. The total water solvate content per formula unit is given as 5.27 by summing up all the site occupation factors contributing to water oxygen atoms; sof(O12) = 0.5, sof(O14) = 1.0, sof(O15) = 1.0, sof(O16) = 0.5, sof(O17) = 0.757, sof(O18A) = sof(O18B) = 0.5, and sof(O19) = 0.5, where the site occupation factor of atom O17 located at a general position was refined and converged at sof(O17) = 0.757. By supposing this site to be a unity in its ideal crystal form before losing the content in air, the hydration number of this orange crystal is tentatively supposed as 5.5 rather than 5.27. The judgment of the binding direction of O and NH of each pivalamidate was not straightforward. As a result of our least-squares analysis carried out for several possible combinations of binding directions, all the calculations resulted in asymmetric features in the temperature factors of N and O atoms of any amidate. Thus, we decided to accept a model in which the two amidate O and NH units are both disordered in two binding directions in an equal probability. We may rationally rule out the coexistence both HH and HT isomers within this geometry due to the reasonably small temperature factors of other atoms comprising the dimeric unit, including the two Pt ions. In this model, the N and O atoms overlapping at the same site is constrained to possess common parameters (coordinates and thermal parameters). This model afforded values of U_{eq}(O1A) = Ueq(N1B) = 0.0360(9), U_{eq}(N1A) = U_{eq}(O1B) = 0.0394(9), U_{eq}(O2A) = U_{eq}(N2B) = 0.0403(9), U_{eq}(N2A) = U_{eq}(O2B) = 0.0362(9). As a result, it can be considered that the HH dimer is itself disordered in two directions in this asymmetric unit, which is quite reasonable considering the fact that one of the adjacent $[Pt(ox)_2]^{2-}$ anions is also disordered over two sites. In the same manner as **3**, refinement of hydrogen atoms does not reach acceptable convergence and therefore all H atoms, except for those of water molecules, were located at their idealized positions (C-H(methyl) = 0.98 Å, C-H(bpy) = 0.95 Å, N-H(pivalamidate) = 0.88 Å; 200 K), and included in the refinement in a riding model approximation, with U_{iso}(methyl H) = 1.5U_{eq}(bonded C), U_{iso}(aromatic H) = 1.2U_{eq}(bonded C), and U_{iso}(H of amidate N-H) = 1.2U_{eq}(bonded N). H atoms of water molecules could not be located. In the final difference Fourier synthesis, the highest peak (1.37 e/Å^3) was located at 0.88 Å of Pt1, while the deepest hole (-0.85 e/Å^3) was located at 0.68 Å from Pt2. In the CheckCIF report, an alert in level A is given to indicate that there is an unusually short interatomic D...A contact at O16…O18B = 2.3547 Å. However, these atoms are located with each having a site occupation factor of 0.5 and therefore there is no bumping problem here.

*Structure Refinement Details of [HH-$Pt_2(bpy)_2(\mu$-pivalamidato$)_2]_2$[$Pt(ox)_2]_2$·8H_2O (**5**)*

The asymmetric unit consists of an HH dimer cation, a $[Pt(ox)_2]^{2-}$ anion, and four water molecules, where one of the water molecules is disordered over two sites; sof(O14) = 0.75 and sof(O15) = 0.25. H atoms of these water molecules were not located. The trimeric unit, given by the stack of the HH dimer cation and the $[Pt(ox)_2]^{2-}$ anion, further correlated to the adjacent trimeric unit through an inversion center. One of the tertiary butyl groups of pivalamidates is treated as being disordered over two sites with an equal population. All non-hydrogen atoms including those in this disordered geometry were refined anisotropically. As for the binding directions of pivalamidates, the reported combinations have been judged to be valid because of the more balanced temperature factors among the N and O atoms within each ligand; U_{eq}(N1) = 0.0453(14), U_{eq}(O1) = 0.0620(14), U_{eq}(O2) = 0.0572(13), U_{eq}(N2) = 0.0491(14). The reverse combination clearly gives an asymmetric feature on these values: U_{eq}(N1 refined as O) = 0.0697(15), U_{eq}(O1 refined as N) = 0.0406(13), U_{eq}(O2 refined as N) = 0.0363(12), and U_{eq}(N2 refined as O) = 0.0733(15). Moreover, the latter combination results in an increase in the final reliability factors (R1[$F^2 > 2\sigma(F^2)$] = 0.0376 and wR2(F^2) = 0.0681; see Supplementary Table 2 for those adopted solution). Similarly, refinement of hydrogen atoms does not reach acceptable convergence and therefore all H atoms, except for those of water molecules, were located at their idealized positions (C-H(methyl) = 0.96 Å, C-H(bpy) = 0.93 Å, N-H(pivalamidate) = 0.86 Å), and included in the refinement in a riding model approximation, with U_{iso}(methyl H) = 1.5U_{eq}(bonded C), U_{iso}(aromatic H) = 1.2U_{eq}(bonded C), and U_{iso}(H of amidate N-H) = 1.2U_{eq}(bonded N). H atoms of water molecules were not located. In the final difference Fourier synthesis, the highest peak (1.14 e/Å^3) was located at 1.10 Å of Pt1, while the deepest hole (-1.07 e/Å^3) was located at 0.46 Å from Pt1.

*Structure Refinement Details of [HT-$Pt_2(bpy)_2(\mu$-pivalamidato$)_2$][$Pt(ox)_2$](NO_3)·7H_2O (**6**)*

Half a part of the formula unit is found in the asymmetric unit of **6**. An inversion center is located at the center of each $[Pt(ox)_2]^{2-}$. On the other hand, a two-fold axis passes through the center of each dinuclear platinum cation, showing that the HT

arrangement of bridging pivalamidates derives from a crystallographic requirement. The binding direction of O and NH of pivalamidate was unambiguously determined as a result of least-squares calculations performed for two possible arrangements of the ligand. The reported combination has been judged to be valid: U_{eq}(O1) = 0.0320(8) and U_{eq}(N1) = 0.0300(9). To the contrary, a reverse combination clearly gives an asymmetric feature on these values: U_{eq}(O1 refined as N) = 0.0143(8) and U_{eq}(N1 refined as O) = 0.0526(11). Moreover, the latter combination leads to dramatic increase in the final reliability factors (R1[$F^2 > 2\sigma(F^2)$] = 0.0259 and wR2(F^2) = 0.0503; see Supplementary Table 2 for those adopted solution).

On the other hand, water molecules and nitrate anions are found to be disordered over several sites along the channel defined with (x, y) = ($^1/_2$, $^1/_2$). Appropriate models were first pursued based on careful examination of difference Fourier maps using the PLATON program, and rigid groups were constructed in the KenX program. As a result, the model shown Supplementary Figure 5 has been judged to best explain the observed electron densities.OOne nitrate anion (N4, O9, O10, and O10*) is located around a two-fold axis, while the other nitrate anion (N5, O11, O12, and O13) is disordered across an inversion center. The situation becomes further complicated due to the overlapping densities at O14 and O15, considered as water oxygen atoms disordered over these geometries. A reasonable model could be attained by supposing three types of structures depicted in figures C-E, where the populations defined by 0.5$\mathbf{P_A}$ and 0.5$\mathbf{P_B}$ respectively correspond to the population of the former N4-O10* nitrate and the latter N5-O13 nitrate in the asymmetric unit. As depicted in figure C, it is considered that the N4-O10* nitrate is hydrogen-bonded to the water molecule O14. Figures D and E also explain how the N5-O13 nitrate forms hydrogen bonds with O12 and O15.

Atoms N4, O9, O10, and O15 were refined with a common isotropic displacement parameter. In the same manner, atoms N5, O11, O12, O13, O14 were refined with a common isotropic displacement parameter. The nitrate anions are fixed as rigid groups; the N-O distances are restrained at N-O = 1.22(1) Å, three O···O distances within each nitrate are restrained as equal, and four atoms of each nitrate are restrained to be planar. As a result, the site occupation factors have been determined as follows: 2 x sof(O11) = 2 x sof(O13) = 2 x sof(N5) = 2 x sof(O15) = sof(O11) = 0.45126 = 0.451 ($\mathbf{P_B}$), and sof(O10) = 2 x sof(N4) = 2 x sof(O9) = sof(O14) = 0.54939 = 0.549 ($\mathbf{P_A}$), where summation of these two separately refined parameters affords 1.00, indicating that nitrate content is one per three Pt atoms. On the other hand, water molecules defined with atoms O6, O7, and O8 do not suffer from any disorder problems, where atom O8 is located on the two-fold axis.

Thus, the contents of nitrate and water molecule per formula unit can be estimated as follows:

(nitrate content per three Pt atoms) = 2 x (nitrate content in the asymmetric unit)
= 2 x sof(N4) + 2 x sof(N5) = (2 x 0.275) + (2 x 0.226) = <u>1.00</u> → **1**

(water content per three Pt atoms) = 2 x (water content in the asymmetric unit)
= 2 x sof(O6) + 2 x sof(O7) + 2 x sof(O8) + 2 x sof(O14)
+ 2 x sof(O15) + 2 x {sof(O12) - sof(O11)}
= (2 x 1) + (2 x 1) + (2 x 0.5) + (2 x 0.54939)
(2 x 0.2256) + (2 x 0.2256)
= <u>7.00</u> → **7**

Thus, the empirical formula of **6** determined crystallographically can be expressed by [Pt(2.33+)$_2$(bpy)$_2$(μ-pivalamidate)$_2$][Pt(2.33+)(ox)$_2$](NO_3)·7H_2O. As a result of our extensive efforts made thus far in the past decade years, we now conclude that the nitrate content must be close to unity based on the following results: (i) elemental analysis (reported in the main text); (ii) X-ray crystallography; (iii) no diffuse scattering has been observed at all for this compound; (iv) the nitrate content determined by XPS based on the integrated intensity ratio of N(nitrate/nitrtrite) 1s and N(bpy) 1s peaks well agrees with this conclusion (Area(nitrate)/Area(bpy) = 1.0/4) (*vide infra*).

All H atoms, except for those of water molecules, are located at their idealized positions (C-H(methyl) = 0.96 Å, C-H(bpy) = 0.93 Å, N-H(pivalamidate) = 0.86 Å), and included in the refinement in a riding model approximation, with U_{iso}(methyl H) = 1.5U_{eq}(bonded C), U_{iso}(aromatic H) = 1.2U_{eq}(bonded C), and U_{iso}(H of amidate N-H) = 1.2U_{eq}(bonded N). H atoms of water molecules were not located. In the final difference Fourier synthesis, three residual peaks in the range 1.18-1.28 e/Å^3 were observed within 1.06 Å of the Pt atoms. The deepest hole (-0.75 e/Å^3) was located at 0.37 Å from O16.

For computations, the water and nitrates were treated explicitly, and a minimum energy state was obtained from a crystallographic starting point.

Supplementary References

1 O'Halloran, T. V.; Lippard, S. J. Isomerization of binuclear amidate-bridged platinum(II) amine complexes: a platinum-195 NMR investigation *Inorg. Chem. 28*, 1289-1295 (**1989**)

2 Sheldrick, G. M. (1996). SADABS. University of Göttingen, Germany

3 Bruker (2001). SAINT (Version 6.22) and SMART (Version 5.625). Bruker AXS Inc., Madison, Wisconsin, USA

4 Sheldrick, G. M. (1997). SHELXS97. University of Göttingen, Germany

5 Sheldrick, G. M. (1997). SHELXL97. University of Göttingen, Germany

6 Sakai, K. (2008). KenX: Program Assisting Structure Refinement in SHELXL-97. Kyushu University, Japan; Downloadable at http://www.scc.kyushu-u.ac.jp/Sakutai/softwares/kenx.zip/

7 Spek, A. L. (1998). PLATON. Multipurpose Crystallographic Tool. Utrecht University, The Netherlands

8 Johnson, C. K. (1976). ORTEPII. Report ORNL-5138. Oak Ridge National Laboratory, Tennessee, USA

9 Molecular Structure Corporation (2001). TEXSAN, Version 1.11r1. Single Crystal Structure Analysis Software. MSC, 3200 Research Forest Drive, The Woodlands, TX 77381, USA

10 Sakai, K.; Shiomi, M.; Tsubomura, T.; Kato, K.; Yokoyama, Y.; Kajiwara, T.; Ito, T. A head-to-head isomer of bis (μ-N-methylisonicotinamidato) bis [cis-diammineplatinum (II)] tetraperchlorate *Acta Cryst. E59*, m559 – m561 (**2003**)

11 Sakai, K.; Kurashima, M.; Akiyama, N.; Satoh, N.; Kajiwara, T.; Ito, T. A head-to-head isomer of di-u-pivalamidato-k4*N*,*O*-bis-[(1,10-phenanthroline-k2*N*,*N*')-platinum(II)] dinitrate dihydrate *Acta Cryst. C59*, m345 – m349 (**2003**)

12 Sakai, K.; Ikuta, Y.; Tsubomura, T.; Kato, K.; Yokoyama, Y.; Kajiwara, T.; Ito, T. A head-to-tail isomer of bis [μ-2-(N′-methyl-4, 4′-bipyridinium-1-yl) acetamidato] bis [cis-diammineplatinum (II)] hexaperchlorate dihydrate *Acta Cryst. E59*, m780 – m783 (**2003**)

13 Yokokawa, K.; Sakai, K. Di-μ-pivalamidato-ϰN:O;O:N-bis[(2,2-bipyridine-ϰN,N)(sulfato-ϰO)platinum(III)] Tetrahydrate in a Head-to-Tail Isomerism. *Acta Cryst. C60*, m244 – m247 (**2004**)
